# Construction, Cloning, and Expression of CagA Recombinant Protein of *Helicobacter pylori*

**Published:** 2020

**Authors:** Abbas Shapouri Moghaddam, Shamseddin Mansouri, Alireza Neshani, Farzaneh Firoozeh, Azade Matinpur, Azad Khaledi, Mehran Ghazalibina

**Affiliations:** 1. Department of Immunology, BuAli Research Institute, Faculty of Medicine, Mashhad University of Medical Sciences, Mashhad, Iran; 2. Antimicrobial Resistance Research Center, Department of Microbiology, Ghaem Hospital, Mashhad University of Medical Sciences, Mashhad, Iran; 3. Department of Microbiology, Faculty of Medicine, Alborz University of Medical Sciences, Karaj, Iran; 4. Infectious Diseases Research Center, Faculty of Medicine, Kashan University of Medical Sciences, Kashan, Iran; 5. Department of Microbiology and Immunology, Faculty of Medicine, Kashan University of Medical Sciences, Kashan, Iran; 6. Department of Microbiology, Faculty of Public Health, Tehran University of Medical Sciences, Tehran, Iran

**Keywords:** CagA, *Helicobacter pylori*, Recombinant proteins, Vaccine candidate

## Abstract

**Background:**

This study aimed to assess construction and expression of CagA recombinant protein of *Helicobacter pylori (H. pylori)* in *Escherichia coli (E. coli)* BL21.

**Methods:**

Bioinformatics was used in designing the desired gene by Gene Runner. Next, the construct was subcloned to pET21b vector and this process was confirmed by Polymerase Chain Reaction (PCR), enzyme digestion and sequencing techniques. Then, it was cloned in the Escherichia coli BL21 as an expression host. Expression of protein was verified using sodium dodecyl sulfate- polyacrylamide gel electrophoresis (SDS-PAGE) and Western blotting technique. For purification of the protein, the Ni-NTA column was used. Protein concentration was determined by the Bicinchoninic Acid Protein Assay Kit (Parstoos). Finally, Western blotting was performed using CagA antibodies and normal human serum for determining immunogenicity feature with human antiserum.

**Results:**

According to the results of the present study, CagA construct was cloned into the pET21b vector and after confirmation and cloning in host expression, recombinant protein with the size of 38 *kDa* was successfully expressed and purified. The recombinant CagA protein showed immunogenicity characteristics with human antiserum.

**Conclusion:**

In conclusion, only 5′-end of recombinant protein CagA with high immunogenicity effects was successfully constructed, cloned and expressed. Also, CagA recombinant protein showed good immunogenicity activity with human antiserum.

## Introduction

Overall, almost 75% of the whole gastric cancers and 63.4% of the total stomach ulcers are caused by *Helicobacter pylori (H. pylori)* 1,2. The most important concern about *H. pylori* is the emergence of drug-resistant strains which necessitate the use of a proper vaccine against pathogenic strains 3,4. So far, several virulence factors have been identified for *H. pylori* 5,6. One of them is CagA that is encoded by *cagA* gene. CagA protein is one of the most immunogenic proteins of *H. pylori* and is also associated with type 1 strains that induce atherosclerosis and heart coronary diseases 7,8.

Most studies used carbonic end of cagA, due to the changes and diversity in its C-terminal fragment in various isolates of *H. pylori*, but the 5′- end of cagA has no usable restriction endonuclease sites as well as EPIYA motif (this region is toxic for host cells) 9 and contains sequences with properties of conserved regions with the high induction power of the immune system (both cellular and humoral arms of immune system, especially cellular arm which is most important in vaccine design) 10,11. Also, the whole protein of CagA is very difficult to express and purify, and due to the antigenic changes, diversity, and disturbance in the folding of the antigen epitopes, the complete fragment of CagA would not be able to stimulate an appropriate immune response 12. On the other hand, the epitopes of its protected regions may not be accessible to the immune system as a result of inappropriate folding 13,14. Therefore, in this study, this antigenic fragment was selected to be investigated.

According to the importance of CagA protein in diseases related to *H. pylori*, this study aimed to assess construction and expression of CagA recombinant protein of *H. pylori* in *Escherichia coli (E. coli)* as an expression host.

## Materials and Methods

### Construction of recombinant plasmid

At first, as mentioned in previous work 1, the sequence of *cagA* gene of *H. pylori* strain 26695 was obtained through searching data banks (NCBI, UniProt, …). Sequence 841 *bp* of 5′-end of cagA was chosen and two enzymes cutting sites of BamHI and SalI (Fermentas, Lituania) were put in 5′ and 3′ ends of cagA fragment. Primer 3 and gene runner tools were used for designing primers. Bioinformatics studies were performed using associated software in silico. Then, the desired fragment was sent to Generay Company (China) for synthesis. DNA construct was cloned into the Multiple Cloning Sites (MCS) of the PGH vector. After that, the *E. coli* strain Top10 transformed. *E. coli* transformation was done on an LB agar plate containing 100 *mg/ml* of ampicillin. PCR, enzyme digestion and sequencing were used for confirmation of transformed colonies. PGH-G plasmids were extracted from *E. coli* (Top10) using extraction plasmid kit (Genet Bio Inc., South Korea) and double digestion was performed using RE enzymes to get cagA fragment based on manufacturer’s directions. To create a recombinant expression vector, double digested cagA fragment was cloned into pET21b. The ligating reaction was used to enter the *cagA* gene into the expression vector of pET21b. The ligation reaction was performed in the presence of T4 DNA ligase enzyme and ligase buffer at 16*°C* overnight. The cloning procedure completed using restriction endonuclease digestion, sequencing (Macrogen Company, South Korea) and PCR of the insert using universal T7-promoter and T7-terminator primers (TAG Copenhage A/S Symbion, Denmark).

### Expression of recombinant protein in *E. coli* BL21 strain

In the expression stage, pET21b/cagA fragment was transformed into the *E. coli* BL21 strain as an expression host. For protein expression, IPTG (Isopropyl β-D-1-thiogalactopyranoside) was used as an inducing material in different concentrations (0.2, 0.5 and 1 *mM*), and then, to get the best time and temperature for induction of cells, different temperatures (4, 16, 18, 28 and 37°*C*) and for incubation, various times (4, 8, 16 and 24 *hr*) were used. Next, collected cells were sonicated 3–4 times with one *min* interval between cycles. Cells were pelleted by use of centrifugation at 14,000x *g* for 15 *min* at 4°*C*. The recombinant protein (CagA) was entered to the inclusion bodies (Insolubility phase) and for dissolving inclusion bodies and denaturation of protein expression in cells (Protein was in the pellet), 6M guanidine HCl was used.

### SDS-PAGE

SDS-PAGE technique was used with a concentration of 12% polyacrylamide gel at voltage of 120 *V* in the presence of the marker to verify the recombinant protein expression. Then, for staining of protein bands, Coomassie Brilliant Blue R250 was applied. Then, band size was judged in comparison to protein marker (Thermo scientific, US) 15.

### Purification of recombinant protein from *E. coli* lysate

Because His-tag was inserted in vector and based on designed primers, nickel affinity chromatography resin (Ni-NTA chromatography) from QIAGEN Company was used for protein purification according to the previous study 16. The purity of the recombinant protein was characterized by the use of SDS-PAGE and Western blotting techniques. Lastly, for the removal of imidazole and other waste constituents, dialysis was performed by PBS buffer (pH=7.5) at 4*°C* overnight. The purity of the recombinant protein was characterized by the use of SDS-PAGE and Western blotting techniques. Bicinchoninic Acid Protein Assay Kit (Parstoos) was used for determination of protein concentration.

### Western blotting technique

In performing Western blotting technique, the separated protein by SDS-PAGE gel was transmitted to PVDF membrane (Amersham) and immunoblotting was completed using anti-poly histidine-peroxidase monoclonal antibody (Sigma-Aldrich). Regarding the standard direction (Manufacturer’s direction), protein bands were finally shown by Chemiluminescent Western Blot Kit (Parstoos). The antibody dilution of 1/7000 was applied in this method 17.

### Patients

Serum was taken from 10 patients referred to the internal ward of Shahid Beheshti hospital, Kashan in 2018. Also, upper gastrointestinal endoscopy, biopsy specimens from the gastric antrum for hematoxylin/eosin staining, and rapid urease test and culture were used for inclusion of patients with active ulcer disease and gastric cancer 2,18.

### Immunogenicity analysis

Western blotting was performed according to the standard protocol as previously described using CagA antibodies and normal human sera as a negative control^4^.

## Results

**Figure 1.**
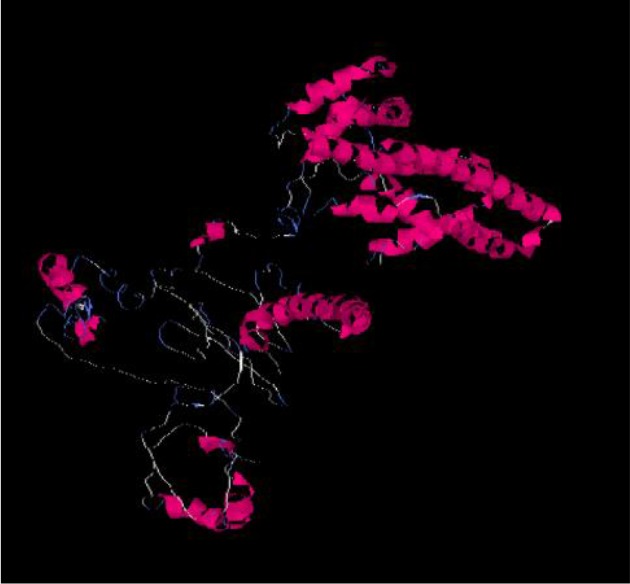
The *three* dimensional *spatial structure* of *cagA* protein using I-TASSER and Discovery Studio Software.

**Figure 2.**
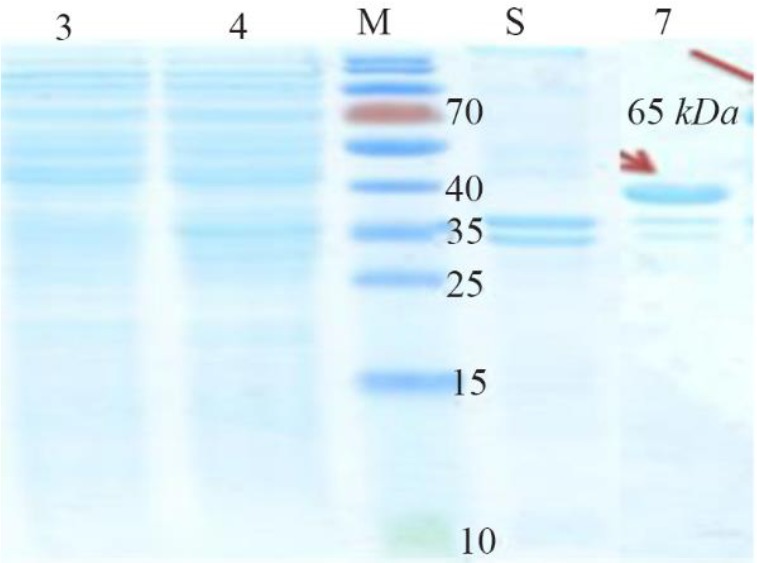
Initial confirmation of CagA protein using SDS-PAGE. Left to right, Lanes 3 and 4 (Supernatant): Lane 3: BL21 non-induced, Lane 4: CagA protein. M: Protein Marker. Lanes 5 and 7 (Precipitate): Lane 5: Non-induced BL21, Lane 7: Protein *CagA*.

**Figure 3.**
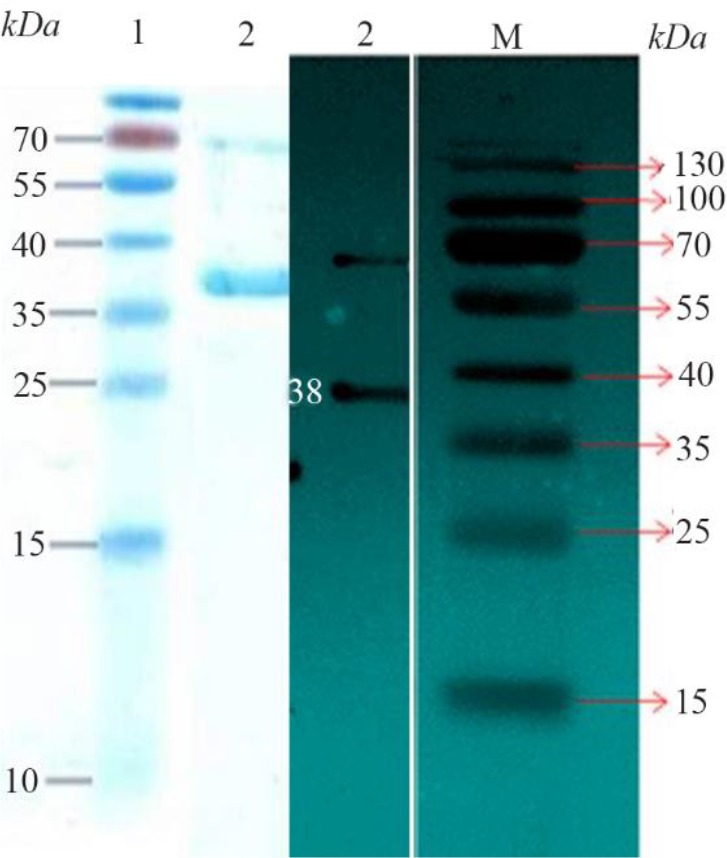
Left picture: SDS PAGE for purified recombinant protein. Lane 1: Protein marker and Lane 2 shows the protein 38 *kDa* (*CagA*). Right picture: Image related to Western blotting technique for confirmation of *CagA* expression. Lane 2: It corresponds to protein 38 *kDa* (*CagA*), and Lane M: Protein marker.

The spatial structure of the *cagA* gene is shown in figure 1. After receiving cagA construction in PGH vector, confirmed by enzyme digestion, two fragments 841 *bp* (cagA) and 2907 *bp* (PGH) were detected on gel agarose. After sub-cloning into the expression vector pET21b, checked by PCR, enzyme digestion (using BamHI and SalI) and two distinct bands 841 *bp* (cagA) and 5442 *bp* (pET21b) were detected and sequencing method was used for definitive confirmation. After that, sub-cloned pET21b (+) fragment was cloned into the expression host (*E. coli* strain BL21). The optimal temperature, IPTG concentration and time were 37*°C* and 0.2 *mM* and overnight, respectively. Because protein was entered into the insolubility phase as mentioned in the methodology section, 6 *M* guanidine HCl was used for dissolving inclusion bodies and denaturation of protein expression in cells. Then, SDS page and Western blotting was carried out for CagA protein (Figures 2 and 3).

### Purification of recombinant protein from *E. coli* lysate

Because the His-tag was inserted in vector and based on designed primers, nickel affinity chromatography resin (Ni-NTA chromatography) from QIAGEN Company was used for protein purification according to the previous study (Figure 3). The Western blotting technique confirmed the immunogenicity of the recombinant CagA protein to human antiserum.

## Discussion

CagA has been introduced as an oncoprotein because it is accompanied with severe pathological results and most patients with gastric cancer are CagA positive; also, its effects on tumor suppressor pathways have been shown 19. For gene expression, Esmaeili *et al* 20 used pET28 a (+) vector which contains T7 promoter and *E. coli*, BL21 (DE3) host to produce recombinant CagA to a considerable amount. In another study, Farjadi *et al* 21 used prokaryotic expression vector pET32a and *E. coli* BL21 (DE3) as expression host. Both studies performed ligation at 16*°C* overnight, and double cut technique was used to clone *cagA* gene and applied antibiotic in both studies was kanamycin and the protein purification was done by nickel affinity chromatography resin (Ni-NTA chromatography) (QIAGEN, USA). Although cloning and expression of CagA protein by Tummuru has also been done previously, but because of its large size (120 *kDa*), experiments like two mentioned studies were performed in this research with the use of pET21b (+) vector and BL21 (DE3); the applied antibiotic in the present study was kanamycin and expression of CagA antigen, in line with Han *et al*’s 10 study, led to the formation of inclusion body which is probably due to differences in the type of expression vector and designing. In previous studies, optimization has not been used but in the current study optimization and codon usage for the desired fragment was applied and consistent codons with expression host *E. coli* BL21 (DE3) was replaced instead of codons of its fragment. Existence in three reading frames, using IPTG as the inducer, also suitability for expression of recombinant proteins due to protease deficiency in BL21 (DE3) are indicated as benefits of all pET vectors and pET 21b has been shown as a commercially optimized vector 22. Advantages of expression of proteins in E. coli include easy and quick function besides low costs 23. CagA is one of the ideal antigens which can be applied for improvement of *H. pylori* vaccines. In our study, a CagA expression system of pET21b-cagA BL21 was successfully constructed, cloned and expressed.

## Conclusion

In conclusion, only 5′-end of recombinant protein CagA with high immunogenicity effects was successfully constructed, cloned and expressed. Also, CagA recombinant protein showed good immunogenicity activity with human antiserum.
